# Supplementary material for: Optimizing automated detection of high frequency oscillations using visual markings does not improve SOZ localization
Source: Clin Neurophysiol. Author manuscript; Available in PMC 2025 Aug 1. (PMC11798584; doi:10.1016/j.clinph.2024.05.010)
Supplement: Supplementary material [file NIHMS2048992-supplement-Supplementary_material.docx]

**Supplementary information**


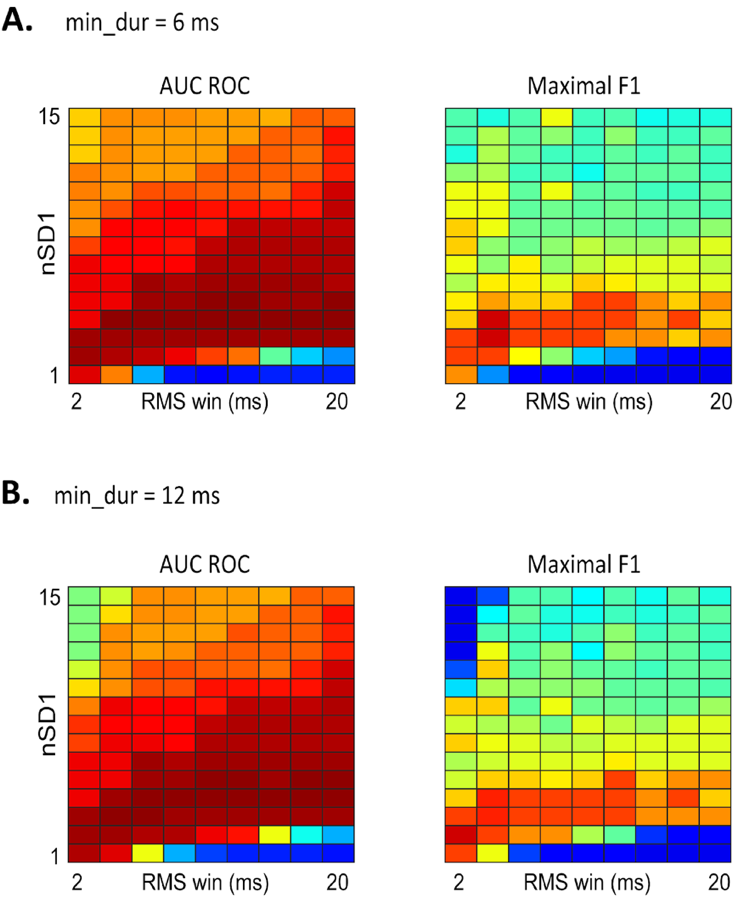


**Supplementary Figure 1.** Minimum event duration does not affect SOZ localization performance. Representative examples of SOZ localization performance across the parameter space, comparing results calculated using minimum event duration of **a** min_dur=6ms and **b** min_dur=12ms. Heatmaps of AUC of the ROC curve and maximal F1 score are shown across the parameter space for patient 5, with RMS window length (RMS_win) varying on the horizontal axis and RMS threshold (nSD1) varying on the vertical axis. All values range from 0 (dark blue) to 1 (dark red), with results closer to 1 indicating good classification of SOZ and nSOZ channels


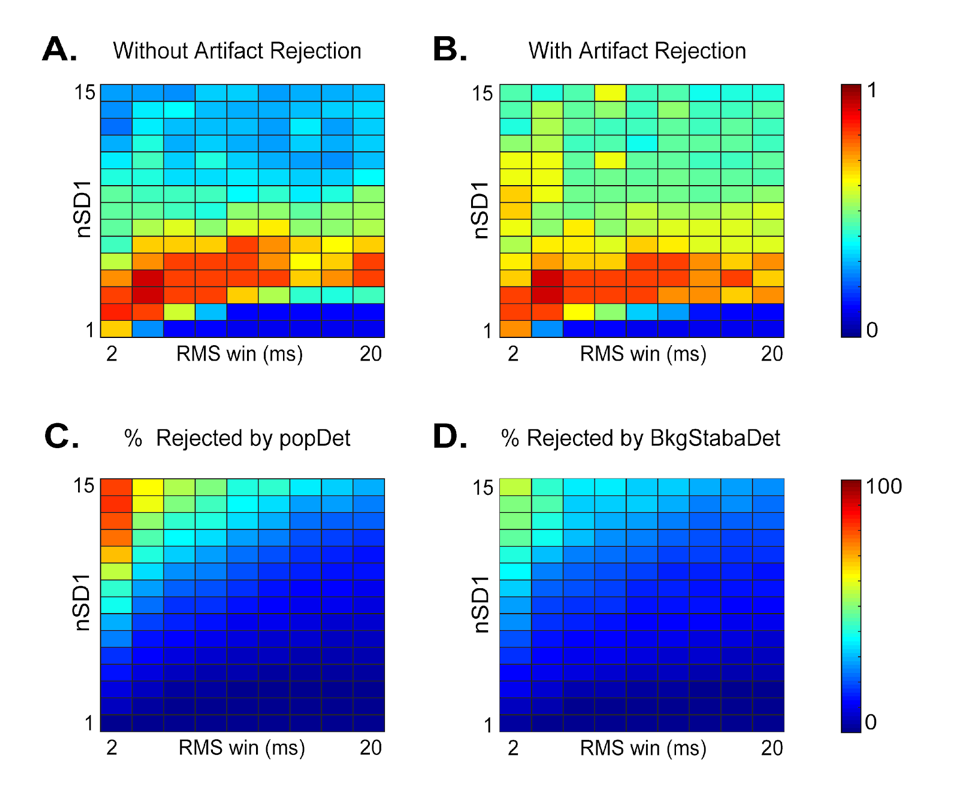


**Supplementary Figure 2.** Artifact rejection does not impact parameters with higher SOZ localization accuracy. Comparison of maximal F1 scores **a** without and **b** with artifact rejection across the parameter space. Percentage of candidate events rejected by **c** PopDet and **d** BkgStabaDet across the parameter space. Results from patient 5 are shown as a representative example.


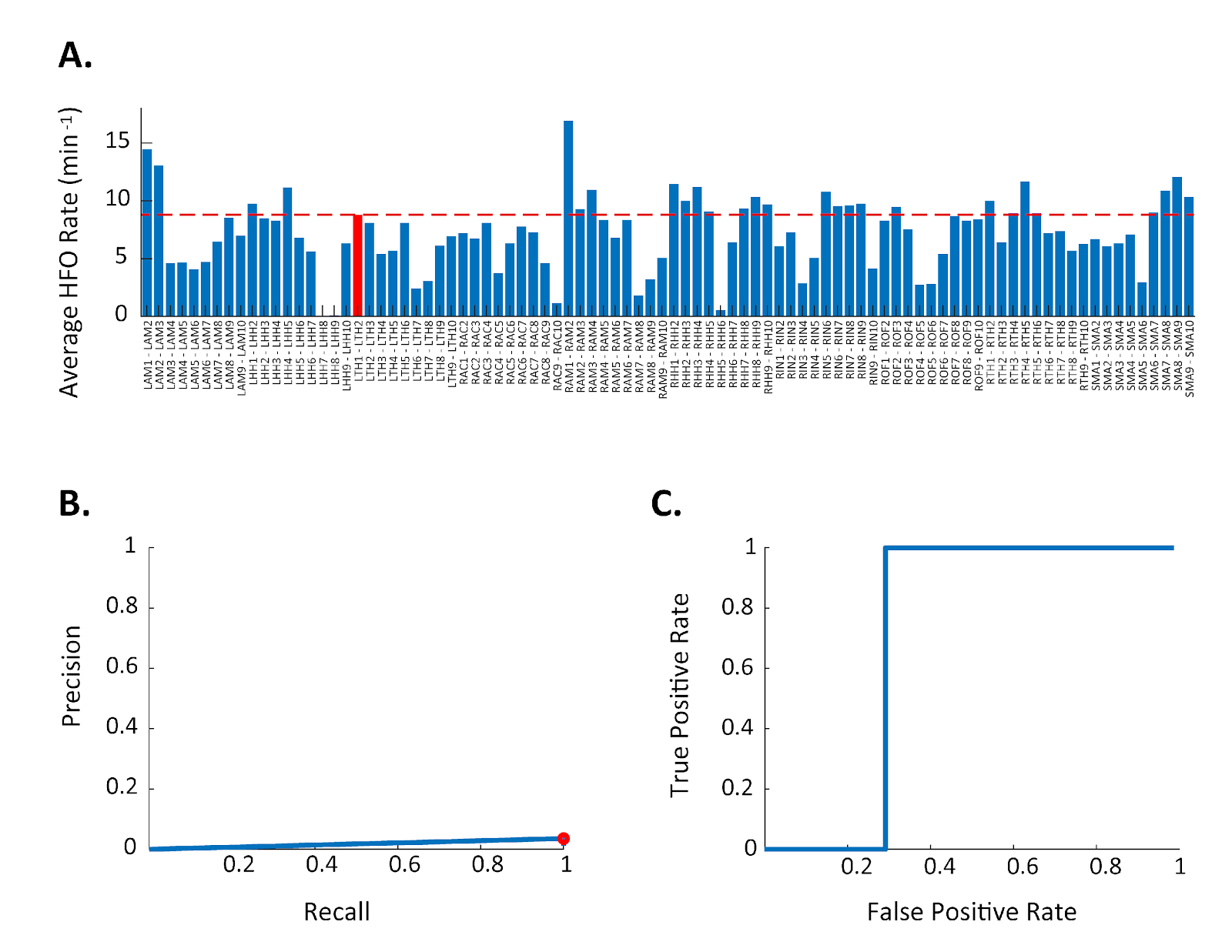


**Supplementary Figure. 3** Imbalanced proportion of SOZ channels and nSOZ channels alters interpretations of precision-recall and receiver-operator-characteristic results. **a** Average HFO rate for each channel in Patient 6 using an RMS threshold of three standard deviations (nSD1=3), RMS window size of 20ms (rms_win=20ms), and minimum event duration of 6ms (min_dur=6ms). In this case, we observe that the HFO rate distribution contains a considerable number of nSOZ channels with high HFO rates. **b** The resulting PR curve with the maximal F1 point circled in red, where Twenty-four nSOZ channels have higher HFO rates than the SOZ channel, thereby resulting in a poor PR curve. **c** The ROC curve shows better results due to the large proportion of nSOZ channels with lower rates compared to the SOZ channel. The low number of SOZ channels (relative to the large number of nSOZ channels) negatively impacts the PR curve when the number of false positives is high, but it minimally impacts the ROC curve.
